# BcRPD3-Mediated Histone Deacetylation Is Involved in Growth and Pathogenicity of *Botrytis cinerea*

**DOI:** 10.3389/fmicb.2020.01832

**Published:** 2020-07-29

**Authors:** Ning Zhang, Zhenzhou Yang, Zhonghua Zhang, Wenxing Liang

**Affiliations:** ^1^Tobacco Research Institute, Chinese Academy of Agricultural Sciences, Qingdao, China; ^2^Key Lab of Integrated Crop Pest Management of Shandong, College of Plant Health and Medicine, Qingdao Agricultural University, Qingdao, China; ^3^Institute of Vegetables and Flowers, Chinese Academy of Agricultural Sciences, Beijing, China

**Keywords:** *Botrytis cinerea*, histone acetylation, deacetylase, pathogenicity, RNA-seq

## Abstract

Histone deacetylase activity plays an important role in transcriptional repression. *Botrytis cinerea* is an important necrotrophic fungal pathogen distributed worldwide and parasites a wide range of hosts. However, the molecular mechanisms of how *B. cinerea* regulates growth and host infection remain largely unknown. Here, the function of BcRPD3, a histone deacetylase of *B. cinerea*, was investigated. Overexpression of the *BcRPD3* gene resulted in significantly decreased acetylation levels of histone H3 and H4. The *BcRPD3* overexpression strains showed slightly delayed vegetative growth, dramatically impaired infection structure formation, oxidative stress response, and virulence. RNA-Seq analysis revealed that enzymatic activity related genes, including 9 genes reported to function as virulence factors, were downregulated in *BcRPD3* overexpression strain. Chromatin immunoprecipitation followed by qPCR confirmed the enrichment of BcRPD3 and H3Kac at the promoter regions of these nine genes. These observations indicated that BcRPD3 regulated the transcription of enzymatic activity related genes by controlling the acetylation level of histones, thereby affecting the vegetative growth, infection structure formation, oxidative stress response, and virulence of *B. cinerea*.

## Introduction

Chromatin remodeling through histone acetylation is an important epigenetic mechanism controlling gene transcription. Histone hyperacetylation results in a loose chromatin structure and transcription activation, while hypoacetylation of histones leads to transcriptional repression by chromatin condensation. The acetylation of histones is antagonistically controlled by histone acetyltransferases (HATs) and histone deacetylases (HDACs) (Lee and Workman, 2007). Characterizing functions of HATs and HDACs is an important way to clarify the roles of histone acetylation.

HDACs are a family of enzymes that reverse lysine acetylation catalyzed by HATs through deacetylation of lysine residues on histones. Except for its important roles in the epigenetic regulation of gene expression, recent studies have uncovered a plethora of non-histone targets of previously known HATs and HDACs, including transcription factors, signaling proteins and other proteins (Emiliani et al., 1998; Bernstein et al., 2000; Yang and Seto, 2008). The acetylation events reversed by HDACs play critical roles for a mass of cellular events, such as protein folding, energy utilization, and cellular metabolism (Imai and Guarente, 2010; Guan and Xiong, 2011). This gene family is conserved in different organisms, comprising of four classes according to sequence homology. Class I, II and IV enzymes are zinc-dependent HDACs. Class III consists of Sir2 or sirtuin family which are NAD^+^ dependent (Gregoretti et al., 2004; Jeon et al., 2014).

RPD3 is the founding member of the Class I HDACs in *Saccharomyces cerevisiae* (Rundlett et al., 1996; Kurdistani and Grunstein, 2003), regulating gene expression through deacetylation of the histones H3 and H4 (Bernstein et al., 2000; Sabet et al., 2004). The genes targeted by RPD3 have diverse functions, including osmotic-stress responsive genes, DNA damage inducible genes, and those involved in determination of replication origin firing (Vogelauer et al., 2002; De Nadal et al., 2004; Sharma et al., 2007).

Filamentous fungi possess RPD3 homologs, playing essential roles in the fungal growth, development and virulence (Lechner et al., 2000; Trojer et al., 2003; Carrozza et al., 2005). For instance, depletion of *RpdA* in *Aspergillus nidulans* leads to a pronounced reduction of growth and sporulation of the fungus (Tribus et al., 2010). In *Aspergillus fumigatus, RpdA* is essential for virulence and loss of RpdA activity results in a lethal phenotype (Bauer et al., 2016, 2019). Hda1 in *Ustilago maydis*, with homology to the yeast RPD3, regulates a subset of genes essential for mature teliospore formation and represses fungal penetration into host epidermal cells (Reichmann et al., 2002; Torreblanca et al., 2003). In *Beauveria bassiana*, RPD3 plays essential roles in regulating transcription of almost all genes in the central development pathway (Cai et al., 2018). *MoRPD3* disruption in *Magnaporthe oryzae* is lethal (Izawa et al., 2009). *Botrytis cinerea*, known as gray mold, can infect over 200 different plant species and is among the most important postharvest fungal pathogens worldwide (Dean et al., 2012). However, there is a lack of reports on the role of RPD3 in *B. cinerea* and its impact on growth and pathogenicity.

Here we investigated the function of BcRPD3 as a histone deacetylase and its impact on fungal development and pathogenesis in *B. cinerea*. Our data revealed that BcRPD3 is involved in the deacetylation of histone H3 and H4. Overexpression of *BcRPD3* exhibited a relatively limited effect on vegetative growth, but dramatically affected infection structure formation, oxidative stress response, and virulence in *B. cinerea*. RNA-seq analysis revealed that enzymatic activity related genes were significantly downregulated in *BcRPD3* overexpression strain compared with wild type, including 9 genes reported to function as virulence factors in *B. cinerea*. Moreover, Chromatin immunoprecipitation followed by qPCR demonstrated that BcRPD3 is recruited to promoter regions of these nine genes, which leads to a decrease of H3Kac abundance at these regions, suggesting an important transcriptional regulatory role of BcRPD3 and H3Kac in *B. cinerea*.

## Materials and Methods

### Strains and Culture Conditions

Wild type B05.10 of *B. cinerea* was used for transformation experiments in this study. All strains were maintained on PDA plates (2% dextrose, 20% potato, and 1.5% agar) or in YPD medium (2% peptone, 1% yeast extract, and 2% glucose). Mycelial growth of the tested strains was measured after 3 days cultivated on PDA plates in 25°C. The conidia number were counted after 10 days incubated on PDA plates. Conidial germination assays were determined as previously described (Liu Y. et al., 2019). Briefly, fresh conidia of wild type, BcRPD3-1, and VRPP-3 after 10 days incubation on PDA plates were harvested in sterilized water and adjusted to the concentration of 2.5 × 10^5^ conidia/ml in PDB (liquid PDA). Twenty microliter of the conidial suspension were dropped onto coverslips and incubated in a moist chamber with a temperature of 25°C and germination rates were determined with over 100 conidia per replicate.

### Generation of *BcRPD3* and *BcRPD3-VRPP* Overexpression Strains

To generate *BcRPD3* and *BcRPD3-VRPP* overexpression construct, CDS of *BcRPD3* and *BcRPD3-VRPP* was cloned into *Nco*I digested pOPT-GFP vector that contains *oliC* promoter, *niaD* terminator, hygromycin phosphotransferase and optimized C-terminal eGFP sequence (Leroch et al., 2011), respectively. The primers used for vector construction are listed in Supplementary Table S1. The constructed plasmids were then transformed into the B05.10 strain using protoplast transformation of *B. cinerea* (Gronover et al., 2001). The resulting transformants were selected by 100 μg/ml hygromycin B and further confirmed by western blot using anti-GFP antibody.

### Assays of Pathogenicity, Infection Cushion Formation, and Sensitivity to H_2_O_2_

Pathogenicity assays were measured as described previously (Yang et al., 2018). In brief, fresh harvested spores of the tested strains were adjusted into same concentration of 5 × 10^5^ conidia/ml in 10 mM glucose, dropped on 3 weeks old tomato leaves and incubated at 25°C for 3 days. Then the lesion diameters caused by different strains with at least five repeats were measured. For infection cushion formation assays, five mm diameter plugs of tested strains cultivated on PDA plates for 3 days were placed on the surfaces of the glass slides and incubated in a moistened chamber at 25°C. After 24, 48, and 60 h post incubation, total number of infection cushions in at least five randomly selected view areas per replicate was examined respectively. To measure sensitivity to H_2_O_2_, same-sized mycelium plugs of different strains were incubated on PDA plates for 3 days supplemented with 0.2% H_2_O_2_ at 25°C and the mycelial growth was then measured.

### RNA Sequencing

The mycelia of wild type B05.10 and BcRPD3-1 strain with three biological replicates were harvested after growth in YPD medium with shaking at 120 rpm for 10 h in 25°C. Total RNA was extracted using the TRIzol reagent according to the instructions of manufacturer. RNA-seq data were analyzed as previously described (Rodenburg et al., 2018). Briefly, Cutadapt (v1.16) software was used to filter the sequencing data. Reference genome index was built by Bowtie2(2.2.6) and the filtered reads were mapping to the reference genome using Tophat2(2.0.14) The mapping statistics were shown in Supplementary Table S2. HTSeq(0.9.1) statistics was used to compare the Read Count values on each gene as the original expression of the gene, and then FPKM was used to standardize the expression. DESeq(1.30.0) was used to analyze the genes of difference expression with screened conditions as follows: an absolute log_2_ value > 1 and *P* < 0.05. All the detected genes were shown in Supplementary Table S3. At the same time, we used R language Pheatmap(1.0.8) software package to perform bi-directional clustering analysis of all different genes of samples. GO categories represented by up- and down-regulated genes were demonstrated using g:Profiler toolset (Raudvere et al., 2019).

### Fluorescent Real-Time qPCR

For qRT-PCR assessment of *BcRPD3* expression during host infection, 50 μL droplets of spore suspension (2 × 10^6^ conidia/mL) of B05.10 were inoculated on the intact leaves of tomato and incubated in 22°C. At 0, 6, 12, 24, 36, and 48 hpi, inoculated leaf samples were collected for RNA extraction. The mycelium was collected after growth in YPD medium with shaking at 180 rpm for 12 h in 25°C. For validation of RNA-seq data, three batches of biological repeats of wild type and BcRPD3-1 were independently collected. RNA was extracted and reverse transcribed using All-In-One RT MasterMix (abm). qRT-PCR was performed using M5 HiPer SYBR Premix EsTaq (Mei5bio). The transcript abundance of candidate genes were calculated using the 2^–△^^Ct^ method. All primers used for qRT-qPCR were listed in Supplementary Table S1.

### Western Blot Assay

The mycelia of the tested strains were grown in YPD at 25°C for 12 h in a shaker. The nuclear proteins were then extracted using Nuclear Protein Extraction Kit (R0050, Solarbio). The obtained proteins were separated by SDS-PAGE and immunoblotted using anti-GFP antibody (ab290, Abcam), anti-H3 antibody (ab1791, Abcam), anti-H4 antibody (07–108, Millipore), anti-H3Kac antibody (06–599, Millipore), anti-H4Kac antibody (06–598, Millipore), anti-H3K9ac antibody (PTM-156, PTM BIO), anti-H3K14ac antibody (PTM-157, PTM BIO), anti-H3K18ac antibody (PTM-158, PTM BIO), and anti-H3K27ac antibody (PTM-160, PTM BIO).

### ChIP-qPCR Analysis

ChIP was performed according to described methods (Liu Z. et al., 2019). Briefly, The mycelia of wild type, GFP containing wild type and BcRPD3-1 strain were harvested after growth in YPD medium with shaking at 180 rpm for 12 h in 25°C. After fresh mycelia had developed, mycelia were cross-linked with 1% formaldehyde (PBS) gently shaking for 25 min and then stopped with glycine with a final concentration of 125 mM for another incubation of 10 min. After cleaning with sterile water for several times, the cultures were frozen and ground with liquid nitrogen. The powder was re-suspended in the lysis buffer (250 mM HEPES pH 7.5, 1 mM EDTA, 150 mM NaCl, 10 mM DTT, 0.1% DeoxyCholate, and 1% Triton) and protease inhibitor cocktail (Roche) with a mycelia/buffer ratio as 0.5 g/2 ml. The DNA was sheared into ∼500 bp fragments using sonicator (Scientz-650E, 35–60% amplitude, ultrasonication for 10 s and stop for 20 s, 15 times). The supernatant was diluted after centrifugation with ChIP dilution buffer (1.1% Triton X-100, 16.7 mM Tris–HCl pH 8.0, 1.2 mM EDTA, 167 mM NaCl). Immunoprecipitation was conducted using 5 μl anti-GFP antibody (ab290, Abcam) or 5 μl anti-H3Kac antibody (06–599, Millipore) together with protein A agarose (Roche) overnight at 4°C. After separation, beads were washed orderly by low-salt wash buffer (150 mM NaCl, 0.2% SDS, 20 mM Tris-HCl PH 8.0, 2 mM EDTA, 0.5% TritonX-100), high salt wash buffer (500 mM NaCl, 2 mM EDTA, 20 mM Tris-HCl PH 8.0, 0.2% SDS, 0.5% TritonX-100), LiCl wash buffer (0.25 M LiCl, 1% Non-idet P-40, 1% sodium deoxycholate, 1 mM EDTA, 10 mM Tris-HCl pH 8.0), and TE buffer. DNA bound to the beads was then eluted and precipitated. ChIP-qPCR was independently repeated three times. Relative enrichment values were calculated by dividing the immunoprecipitated DNA by the input DNA. Primers using for ChIP-qPCR were designed near putative TSS (transcription start site) and listed in Supplementary Table S1.

## Results

### Effect of *BcRPD3* Overexpression on H3 and H4 Deacetylation

**FIGURE 1:**
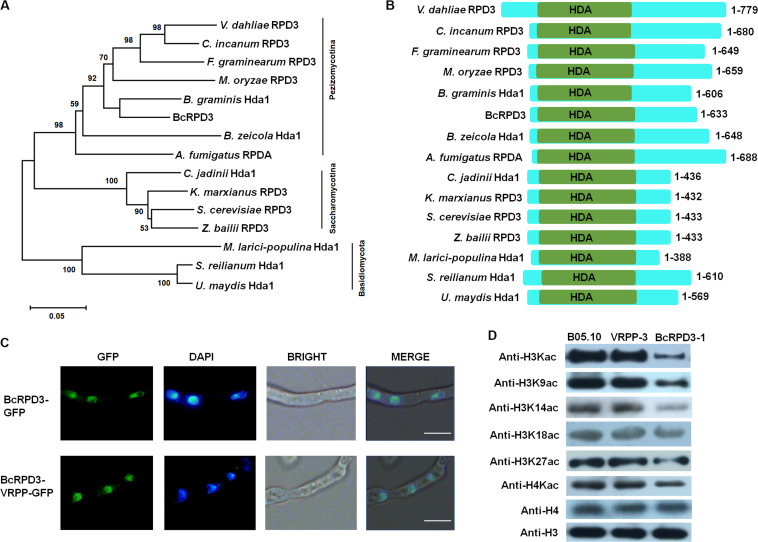
Classification and localization of BcRPD3. **(A)** Phylogenetic tree of BcRPD3 based on a neighbor-joining analysis using MEGA-X. The orthologs are from *V. dahliae* RPD3 (Ensembl: PNH36023), *C. incanum* RPD3 (Ensembl: KZL80576), *F. graminearum* RPD3 (Ensembl: ESU06006), *M. oryzae* RPD3 (Ensembl: MGG_05857T0), *B. graminis* Hda1 (Ensembl: BLGH_01902), *B. zeicola* HDA1 (Ensembl: EUC28153), *A. fumigatus* RPDA (Ensembl: OXN03576), *C. jadinii* Hda1 (Ensembl: ODV71565), *K. marxianus* RPD3 (Ensembl: BAO39086), *S. cerevisiae* RPD3 (Ensembl: KZV08295), *Z. bailii* RPD3 (Ensembl: SJM84961), *M. larici-populina* Hda1 (Ensembl: EGG09787), *S. reilianum* Hda1 (Ensembl: CBQ72606), and *U. maydis* HDA1 (Ensembl: KIS69526). **(B)** Schematic representation of different RPD3 orthologs including their catalytic domains. HDA, histone deacetylase domain. **(C)** Mycelia images for subcellular localization of the GFP-tagged BcRPD3 and BcRPD3-VRPP. Bars = 10 μm. **(D)** Western blotting for the expression levels of histone H4, H3 and acetylation levels of H4, H3 and its Kac sites in wild type B05.10, VRPP-3, and BcRPD3-1 strains.

To identify RPD3 in *B. cinerea*, we used the amino acid sequence of yeast RPD3 protein as query to search against the *B. cinerea* database at EnsemblFungi. As a result, the gene *BcRPD3* (Ensembl: Bcin05g02590) was identified, which encodes a 633 amino acid protein with a DNA sequence of 3234 bp with three introns. A phylogenetic tree of fungal RPD3 orthologs was constructed (Figure 1A). Fungal orthologs of RPD3 are found among necrotrophic fungi (*Botrytis cinerea*, *Colletotrichum incanum*, *Aspergillus fumigatus*), biotrophic fungi (*Blumeria graminis*, *Melampsora larici-populina*, *Sporisorium reilianum, Bipolaris zeicola, Magnaporthe oryzae, Fusarium graminearum, Ustilago maydis*), and yeasts (*Cyberlindnera jadinii*, *Kluyveromyces marxianus*, *Zygosaccharomyces bailii*, *Saccharomyces cerevisiae*). These orthologs clustered into three distinct groups: Pezizomycotina, Saccharomycotina, and Basidiomycota, and all orthologs contained a HDA deacetylase domain (Figure 1B).

qRT-PCR was used to assess the expression of *BcRPD3* during plant infection and vegetative growth phase (mycelium). As shown in Supplementary Figure S1, expression of *BcRPD3* decreased during infectious growth stages, while in mycelium, the expression was elevated and significantly higher than in conidia (0 h). This transcription pattern implicated that *BcRPD3* was more active during vegetative growth phase than during plant infectious stages in *B. cinerea*, indicating that *BcRPD3* may play different roles during multiple growth conditions and developmental stages.

To elucidate the functions of *BcRPD3*, we first tried to delete the *BcRPD3* gene. However, despite numerous attempts (over 200 transformants), we failed to gain *BcRPD3* knock out mutants, indicating that *BcRPD3* plays an essential role for the survival of *B. cinerea*. Alternatively, we generated overexpression strains of *BcRPD3* with a *oliC* promoter and C-terminal GFP in *B. cinerea*. Previous studies showed that swapping the amino acids AGG in HDAC1 (human homolog of BcRPD3) with VRPP which is unique amino acid sequence in class II HDACs, resulted in a loss-of-function mutation of HDAC activity (Wei et al., 2017). We used the amino acid sequences of class I and class II HDACs in human to search against the *B. cinerea* database. As a result, BcRPD3 and BcHos2 (Bcin01g03610) were identified as class I HDACs, while BcHda1 (Bcin15g02130) and BcHos1 (Bcin12g01310) was identified as class II HDACs in *B. cinerea* (Supplementary Figure S2A). Accordingly, we generated *BcRPD3-VRPP* overexpression strains to serve as a silencing transformant of impaired deacetylase activity. A total of 3 transformants were obtained for *BcRPD3* and *BcRPD3-VRPP* overexpression, respectively. We used qRT-PCR to assess the expression level of *BcRPD3* in different strains and the results indicated that *BcRPD3* transcript showed marked up-regulation in overexpression lines compared with B05.10 (Supplementary Figure S2B). In addition, these 3 transformants of each group had the same phenotypes and western analysis with anti-GFP showed indistinguishable protein expression levels (Supplementary Figure S2C). Therefore, only data for transformants BcRPD3-GFP-1 (BcRPD3-1) and BcRPD3-GFP-VRPP-3 (VRPP-3) were presented below.

Fluorescence microscopy was used to observe the transformed mycelia after growth on PDA plates for 2 days. Both of the green signals of the GFP-tagged BcRPD3 and BcRPD3-VRPP overlapped with nuclei stained with DAPI (Figure 1C), indicating BcRPD3 and BcRPD3-VRPP are both mainly located in nucleus. Since RPD3 orthologs reportedly target the acetylation of histones in yeast and filamentous fungus *A. nidulans* (Rundlett et al., 1998; Graessle et al., 2000; Chang and Pillus, 2009; Tribus et al., 2010), protein extracts of wild type B05.10, strain BcRPD3-1 and VRPP-3 were subjected to immunoblotting using acetyl-histone H3, H4 antibodies. The results showed that the acetylation levels of both H3 and H4 significantly decreased in strain BcRPD3-1 compared with wild type, while BcRPD3-VRPP was hardly to detect any significant HDAC activity (Figure 1D and Supplementary Figure S2D). Furthermore, strain BcRPD3-1 exhibited hypoacetylation of histone H3 at K9, K14, and K27 in comparison to the blots of wild type and strain VRPP-3 (Figure 1D). The altered Kac events indicated an ability for BcRPD3 to deacetylate the Kac sites of H3 and H4 in *B. cinerea.*

### Impact of *BcRPD3* Overexpression on Radial Growth, Sporulation, and Conidial Germination

**FIGURE 2:**
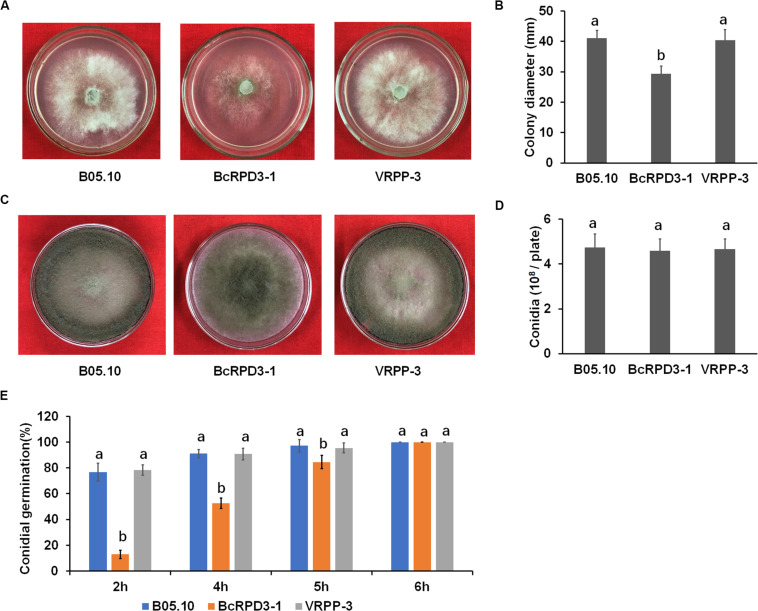
Effects of *BcRPD3* overexpression on mycelial growth, sporulation and conidial germination. The bars represent standard errors from three replicates, and statistical tests were conducted using Tukey’s test for multiple comparisons. Values on the bars followed by different letters are significantly different at *p* < 0.05. **(A)** Mycelial growth of B05.10, BcRPD3-1 and VRPP-3 strains on PDA plates after 3 days of cultivation. **(B)** Quantification of colony diameter of the indicated strains grown on PDA plates for 3 days. **(C)** Conidiation of different strains on PDA after 10 days of cultivation. **(D)** Quantification of conidia produced by the indicated strains on PDA plates (diameter, 4 cm). **(E)** Quantification of the conidial germination of the indicated strains during a time course (6 h) of germination in PDB on glass slides.

To determine whether *BcRPD3* is involved in *B. cinerea* growth, we examined mycelial growth of wild type, BcRPD3-1, and VRPP-3 strains. Our data demonstrated that strain BcRPD3-1 exhibited significant but limited radial growth decrease (around 25%) as compared with the wild type, while strain VRPP-3 didn’t show any significant difference with wild type (Figures 2A,B). To test whether *BcRPD3* played a role in *B. cinerea* conidiation, we determined conidiation ability of tested strains after 10 days inoculated on PDA plates. Our results demonstrated that conidiation of the BcRPD3-1 strain was almost same as wild type and VRPP-3 strains (Figures 2C,D). To determine the viability of conidia produced by the *BcRPD3* overexpression, we observed conidial germination of the conidia harvested from 12-day incubated plates. Our data indicated that overexpression of *BcRPD3* delayed conidial germination on glass slides (Figure 2E). The above data indicated that overexpression of *BcRPD3* slightly affected *B. cinerea* vegetative growth and germination.

### *BcRPD3* Overexpression Affects Virulence, Infection Structure Formation, and Oxidative Stress Response of *B. cinerea*

**FIGURE 3:**
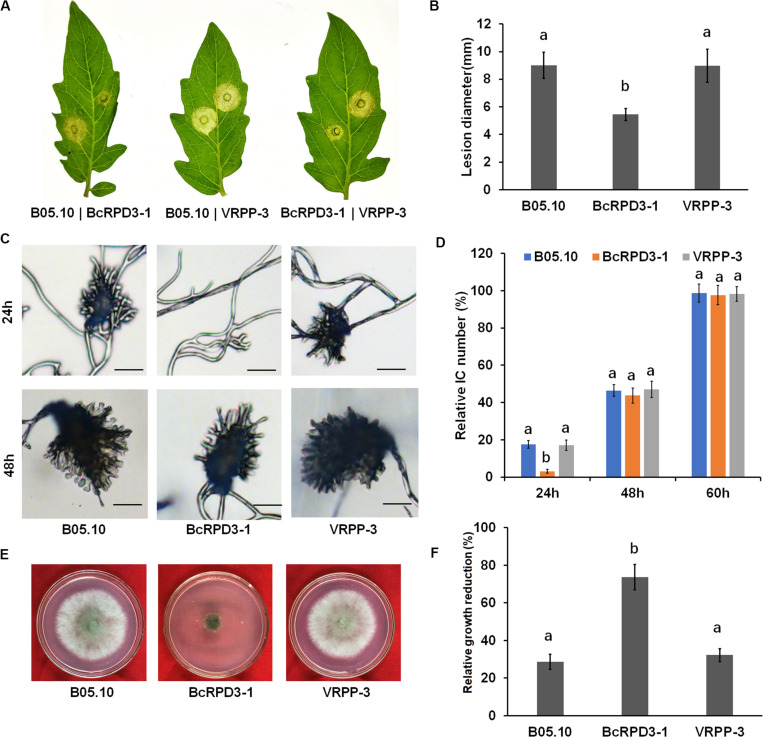
Impact of *BcRPD3* overexpression on virulence, infection cushion formation and oxidative stress response. The bars represent standard errors from five replicates, and statistical tests were conducted using Tukey’s test for multiple comparisons. Values on the bars followed by different letters are significantly different at *p* < 0.05. **(A)** Pathogenicity on tomato leaves after 3 days of incubation. **(B)** Quantification of disease lesions on tomato leaves 3 days after inoculation. **(C)** Infection cushion formation of the indicated strains after 24 and 48 h of incubation on glass slides. Bars = 20 μm. **(D)** Quantification of infection cushion (IC) numbers produced by the indicated strains over a time course. **(E)** Sensitivity of the indicated strains after cultivation on PDA plates supplemented with 0.2% H_2_O_2_ for 3 days. **(F)** Relative growth reduction of the indicated strains which represented the reduction of colony diameters between normal and stress conditions normalized to colony diameters in normal condition.

To investigate the function of *BcRPD3* in *B. cinerea* pathogenicity, an infection assay on tomato leaves was performed. Two days after inoculation, spreading lesions caused by BcRPD3-1 strain was significantly less than wild type and VRPP-3 strain, which is almost reaching 50% (Figures 3A,B). To evaluate the effect of *BcRPD3* on infection structure formation, we observed infection cushion formation of the tested strains. Our data demonstrated that compared with wild type and VRPP-3 strain, BcRPD3-1 strain significantly formed less infection cushions after 24-h incubation and resumed after 48-h incubation on glass slides (Figures 3C,D), indicating that formation of infection cushions was delayed by *BcRPD3* overexpression.

Since sensitivity to oxidative stress is associated with loss of pathogenicity of *B. cinerea* (Yang et al., 2018), we investigated the sensitivity of hyphae of tested strains to oxidative stress. After growth on PDA plates supplemented with 0.2% H_2_O_2_ for 3 days, the relative growth reduction of colony diameter achieved by BcRPD3-1 strain was almost twice as wild type and VRPP-3 strains (Figures 3E,F). Taken together, these data demonstrated that overexpression of *BcRPD3* has negative effects on mediating infection cushion formation and resistance to oxygen stress, which may be partially responsible for the virulence-attenuation of the pathogen overexpressing *BcRPD3*.

### *BcRPD3* Overexpression Downregulated Expression of Enzymatic Activity Related Genes

**FIGURE 4:**
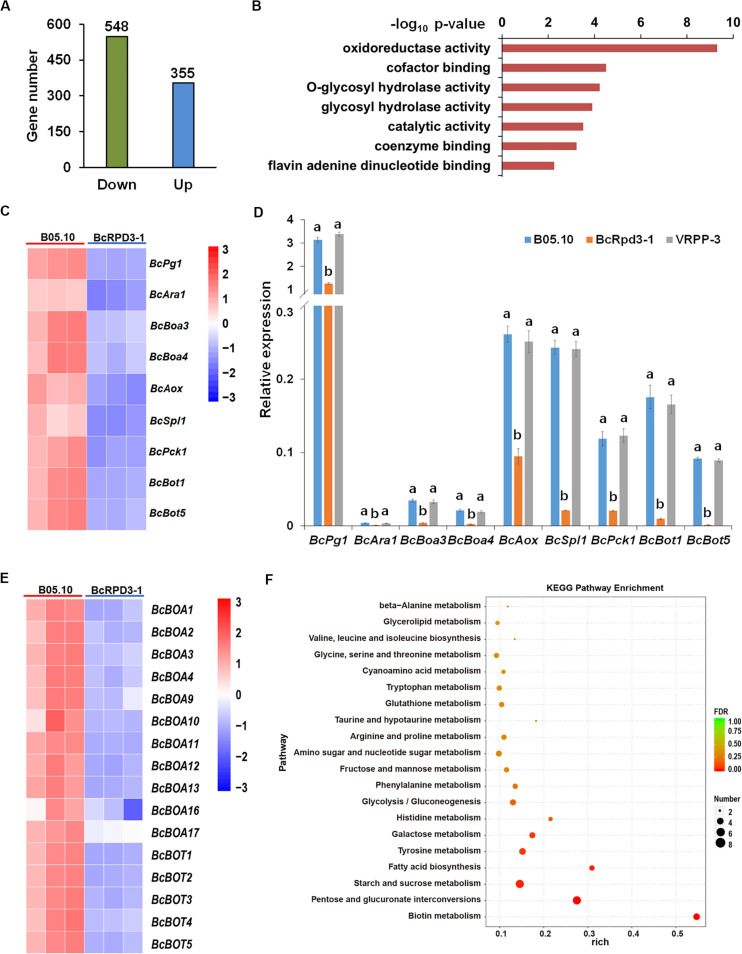
RNA-Seq Analysis of *BcRPD3* overexpression strain. **(A)** Numbers of up- and down regulated genes (*P*-value < 0.05, fold change > 2) in BcRPD3-1 strain compared with wild type B05.10. **(B)** Distribution of functional classification of downregulated genes in BcRPD3-1. Histograms indicate *P-*values of the enriched functional categories. **(C)** RNA-seq analysis of 9 downregulated genes involved in enzymatic activity. Differential expression in three biological replicates is illustrated using a heat map with colored squares indicating the range of expression levels referred to log2 FPKM value. **(D)** qRT-PCR validation of downregulated genes. Expression levels were normalized to β*-tubulin* gene. The bars represent standard errors from three replicates, and statistical tests were conducted using Tukey’s test for multiple comparisons. Values on the bars followed by different letters are significantly different at *p* < 0.05. **(E)** RNA-seq analysis of *BcBoa* and *BcBot* family genes. Differential expression in three biological replicates is illustrated using a heat map with colored squares indicating the range of expression levels referred to log2 FPKM value. **(F)** KEGG pathway enrichment of differentially expressed genes in BcRPD3-1 compared with wild type.

Previous studies have demonstrated that RPD3 is localized in the nucleus and can catalyze deacetylation of histone H3 and H4 in *S. cerevisiae* and filamentous fungus *A. nidulans* (Rundlett et al., 1998; Graessle et al., 2000; Chang and Pillus, 2009; Tribus et al., 2010). As shown in Figure 1D, *BcRPD3* overexpression significantly reduced the acetylation levels of H3 and H4. Therefore, we performed an RNA-Seq analysis to detect genes that might exhibit changes in regulation affected by BcRPD3 in *B. cinerea*. Three biological replicates with mRNA isolated from wild type B05.10 and BcRPD3-1 strain were performed. As a result, 548 down- and 355 upregulated (fold change > 2, *P* < 0.05) genes were identified in the strain BcRPD3-1 compared with B05.10 (Figure 4A and Supplementary Table S3).

Given the function of histone deacetylases in gene repression, we supposed that the target genes of BcRPD3 were likely to be among the downregulated genes. Functional annotation of Gene Ontology (GO) analysis of those downregulated genes revealed that genes involved in enzymatic activity, including oxidoreductase, cofactor binding, glycosyl hydrolase, and catalytic activity were significantly enriched (Figure 4B). Nine downregulated genes (Figure 4C) from representative categories were selected for experimental validation. These 9 genes were reported to be involved in pathogenicity of *B. cinerea*, including oxidoreductase activity-related *BcAox* (alternative oxidase) (Lin et al., 2019), cofactor biding-related *BcBoa3* and *BcBoa4* (botcinic acid biosynthetic genes) (Dalmais et al., 2011), glycosyl hydrolase activity-related *BcPg1* (endo-polygalacturonase) (Have et al., 1998), and *BcAra1* (endo-arabinanase) (Nafisi et al., 2014), catalytic activity-related *BcBot1*, *BcBot5* (botrydial biosynthetic genes) (Dalmais et al., 2011), and *BcPck1* (phosphoenolpyruvate carboxykinase) (Liu et al., 2018), and *BcSpl1* (cerato-platanin family protein) (Frías et al., 2011). qRT-PCR analysis indicated that all of these 9 genes were indeed downregulated in strain BcRPD3-1 compared with B05.10 (Figure 4D). Besides, it is noteworthy that a large proportion of *BcBoa* and *BcBot* family genes regulating synthesis of two groups of non-specific phytotoxins, botrydial and botcinic acid, were dramatically downregulated (Figure 4E), which is in accord with repeated reports that interference with KDAC function leads to altered and co-regulated expression of biosynthetic gene cluster genes (Shwab et al., 2007; Pfannenstiel et al., 2018; Pidroni et al., 2018).

For the up-regulated genes, GO analysis demonstrated that they were involved in iron ion binding, oxidoreductase activity, heme binding, tetrapyrrole binding, and cofactor binding (Supplementary Figure S3A), which exhibiting a prominent characteristic of binding function. Heatmap and qPCR validation of the most significantly up-regulated genes are shown in Supplementary Figures S3B,C. Further KEGG pathway enrichment of differentially expressed genes showed that they were involved in multiple nutrients metabolisms, such as pentose, sucrose, galactose, tyrosine, and histidine (Figure 4F), indicating BcRPD3 may play a role in regulating fungal vegetative growth and development.

### Overexpression of BcRPD3 Results in Increased BcRPD3 and Decreased H3Kac Levels on Promoters of Pathogenicity-Related Genes

**FIGURE 5:**
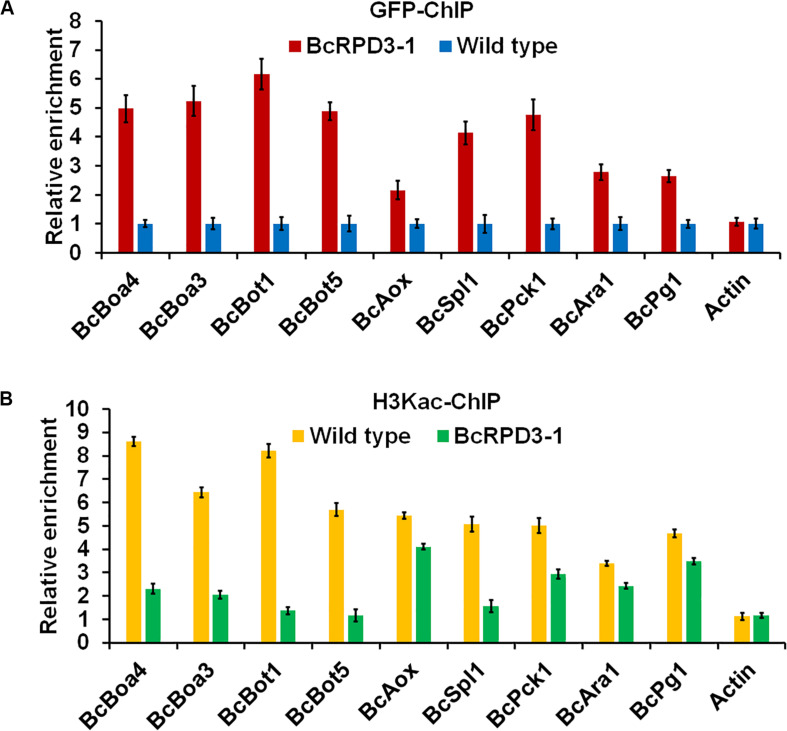
Relative enrichment levels of immunoprecipitated promoter regions of enzymatic activity related genes. The fold enrichment was normalized to input and internal control gene (β*-tubulin*-p). Data are means ± SD (*n* = 3). **(A)** Relative levels of the immunoprecipitated chromatin using anti-GFP antibody from the BcRPD3-1 strain and wild type containing GFP alone. **(B)** Relative levels of the immunoprecipitated DNA using anti-H3Kac from wild type containing GFP alone and BcRPD3-1 strain.

BcRPD3 significantly affected the acetylation level of H3Kac (Figure 1D), and overexpression of *BcRPD3* dramatically reduced the expression levels of 9 pathogenicity related genes (Figure 4D). To investigate whether BcRPD3 directly regulated these genes, a selective chromatin immunoprecipitation (ChIP)-qPCR assay using anti-GFP was used to cross-link DNA target fragments that bind BcRPD3 *in vivo*. Primers in promoter regions near putative TSS (transcription start site) were designed to evaluate enrichment levels of BcRPD3 in the 9 pathogenicity related genes. The results showed that these regions were highly enriched by BcRPD3 in BcRPD3-1 strain compared with wild type strain containing GFP alone (Figure 5A). *ACTIN* was used as a negative reference gene, and the *ACTIN* sequence was not enriched (Figure 5A).

To test whether these promoter regions were also H3Kac locations in genomic DNA, we further performed ChIP using anti-H3Kac antibody followed by qPCR. As shown in Figure 5B, these regions were also enriched by H3Kac in wild type compared with BcRPD3-1 strain, without enrichment in promoter region of *ACTIN* (Figure 5B). These observations demonstrated that BcRPD3 and H3Kac could enrich the promoter regions of 9 pathogenicity related genes, indicating that BcRPD3 and H3Kac participated in gene transcriptional regulation in *B. cinerea*.

## Discussion

RPD3 and its homologs (HDAC1-3) are widely conserved across different organisms. The roles of HDAC1-3 and yeast RPD3 have been well investigated in human diseases such as cancer (Spurling et al., 2008; Hayashi et al., 2010; Müller et al., 2013), regulation of histone acetylation in yeast (Rundlett et al., 1996; Kurdistani and Grunstein, 2003; Yang and Seto, 2008), respectively. In filamentous fungi, several studies have discovered that RPD3 complexes were involved in virulence in *M. oryzae, F. pseudograminearum, A. fumigatus*, and so on (He et al., 2018; Bauer et al., 2019; Zhang et al., 2020). In this study, we investigated the roles of BcRPD3 as a class I HDAC in fungal growth and pathogenesis in order to bridge this knowledge gap in *B. cinerea*.

We first tried to delete *BcRPD3* gene to investigate its function. However, although deletion of *RPD3* is not lethal in *S. cerevisiae*, we failed to obtain knockout mutants after screening over 200 transformants from at least five independent transformations. In fact, many attempts of *RPD3* disruption in filamentous fungi was unsuccessful, as in the case with *Fusarium fujikuroi*, *M. oryzae*, and *A. nidulans* (Izawa et al., 2009; Tribus et al., 2010; Studt et al., 2013; Bauer et al., 2016), suggesting that *RPD3* is an essential gene in filamentous fungi. As an alternative, we generated overexpressed strains of *BcRPD3*. Overexpression of *BcRPD3* dramatically decreased the global acetylation levels of H3 and H4 (Figure 1D). Together with nuclear localization and conserved domain architecture, the result strongly suggests that BcRPD3 is a bona fide histone deacetylase in *B. cinerea*.

Previous reports have shown that class I HDACs function as members of protein complexes *in vitro*. For example, in filamentous fungi, RpdA interacted with RcLS2F in *A. nidulans* (Bauer et al., 2020). Rpd3L-like HDAC complex in *Fusarium Pseudograminearum* contained multiple partners including FpDep1, FpSds3, FpSin3, FpRpd3, FpRxt3, FpCti6, FpPho23, and FpUme6 (Zhang et al., 2020). However, overexpression of RpdA in *Aspergillus sp.* did not cause any phenotypic effects. This has been attributed to the lack of complex partners despite overexpression of the enzyme and is in line with the observation that significant *in vitro* class 1 HDAC activity is only observed for enzyme complexes. From our transcriptomics data, the expression of these putative interaction partners of RPD3 did not show any significant difference between BcRPD3-1 and wild type, indicating that BcRPD3 acting at least in part on its own and this might indicate a different feature of BcRPD3 when compared to its orthologs of other filamentous fungal subclades.

*HDACs* gene family is conserved in different organisms ranging from humans to yeast (Gregoretti et al., 2004). In humans, swapping the amino acids AGG in HDAC1 with VRPP generated a novel HDAC1 mutant with impaired HDAC activity (Wei et al., 2017). In some cases, excessive protein expression may be toxic for organisms, resulting in damages and dysfunction. To test that possibility, we overexpressed BcRPD3-VRPP with a C-terminal GFP in *B. cinerea*. Following western analysis using anti-H3Kac and H4Kac showed that BcRPD3-VRPP protein didn’t possess any HDAC activity (Figure 1D), indicating a conserved function of VRPP mutation for class I HDACs in *B. cinerea*. Therefore, we used *BcRPD3-VRPP* overexpression strain (VRPP-3) as a silencing transformant and our data demonstrated that strain VRPP-3 with almost same protein expression of strain BcRPD3-1 did not show any impaired phenotype compared with wild type. It means that the changed phenotypes of strain BcRPD3-1 are caused by the reinforced HDAC function, but not excessive protein expression.

Overexpression of BcRPD3 did not affect sporulation, but diminished mycelial growth and germination slightly, pointing toward roles of BcRPD3 in regulating fungal vegetative growth and development. Our RNA-seq analysis suggests that global changes of genes involved in multiple nutrients metabolisms, such as pentose, sucrose, galactose, tyrosine, and histidine, is likely to underlie this growth defect (Figure 4F). The results suggest that the overexpression strain probably cannot properly express genes with function in the efficient transport and use of those nutrients due to excess of BcRPD3-mediated histone deacetylation.

Other than vegetative growth, we observed sharp declines in the virulence, infection cushion formation and oxidative stress response in the overexpression strain. As shown in Supplementary Figure S1, expression of *BcRPD3* elevated in mycelium compared with in conidia (0 h) and decreased during infectious growth stages. We speculated that during vegetative growth phase, the expression of endogenous *BcRPD3* in *B. cinerea* was active and kept in a high level which is enough for BcRPD3 to perform its functions, so that excess BcRPD3 may only have limited effects on the vegetative growth. While during infectious stages, expression of *BcRPD3* decreased dramatically and stayed in a low level. Overexpression of *BcRPD3* may break that native regulatory pathway and impaired the process of host infection. Therefore, we presumed that overexpression of *BcRPD3* may play a more important role during the infectious stages than vegetative growth phase. Further RNA-seq analysis showed that 548 genes were significantly downregulated in BcRPD3-1 compared with wild type, including 9 pathogenicity related genes reported to act as virulence factors of *B. cinerea* (Figures 4C,D). Among these, BcPg1, acting as endopolygalacturonase, can converse host tissue into fungal biomass and is required for full virulence (Have et al., 1998). Deletion of phosphoenolpyruvate carboxykinase gene *BcPck1* resulted in slow conidium germination, and delayed infection structure formation (Liu et al., 2018). Alternative oxidase gene *BcAox* is involved in regulation of virulence and oxygen stress response of *B. cinerea* (Lin et al., 2019). *BcAra1* encoding α-1,5-L-endo-arabinanase plays an important role during the infection of host (Nafisi et al., 2014). BcSpl1 is one of the most abundant proteins in the secretome of *Botrytis cinerea* and the knock out mutants showed decreased virulence in a variety of hosts (Frías et al., 2011). The double mutants of *BcBoa* and *BcBot* that do not produce botcinic acid or botrydial displayed markedly reduced virulence (Dalmais et al., 2011). Collectively, together with ChIP-qPCR results, BcRPD3 may directly regulate these 9 pathogenicity related genes to modulate pathogenicity of *B. cinerea*. Furthermore, as the acetylation levels of both H3 and H4 decreased in strain BcRPD3-1, future studies are necessary to explore the regulatory role of H4Kac in *BcRPD3* overexpression strain.

It is reported that deletion of *RPD3* resulted in impaired growth and virulence in several fungi (Reichmann et al., 2002; Bauer et al., 2016, 2019; Cai et al., 2018). Based on our results, enhanced BcRPD3-mediated histone deacetylation also led to diminished mycelial growth, germination, and virulence. Therefore, we speculated that maintaining an appropriate RPD3 level is critical for the survival and pathogenicity of fungal pathogens.

## Conclusion

In conclusion, our data indicate that BcRPD3 is a conserved epigenetic component responsible for histone acetylation and the elevated HDAC activity is important for growth and pathogenicity of *B. cinerea*. Transcriptome data provide enzymatic activity related genes downregulated following BcRPD3 overexpression. The promoter regions of 9 of these genes which were reported participating fungal pathogenicity were shown to be significantly enriched by BcRPD3 and H3Kac. Further illustration of target genes would help further investigate novel control strategy for *B. cinerea*.

## Data Availability Statement

The original contributions presented in the study are publicly available. This data can be found here: https://www.ncbi.nlm.nih.gov. Bioproject ID: PRJNA627027.

## Author Contributions

NZ and WL generated the hypothesis, planned the experiments, and wrote the manuscript. NZ, ZY, and ZZ performed the experiments. All other authors provided comments on the manuscript.

## Conflict of Interest

The authors declare that the research was conducted in the absence of any commercial or financial relationships that could be construed as a potential conflict of interest.

## Supplementary Material

FIGURE S1 Expression profile of *BcRPD3* in mycelium and during host infection by the pathogen. Expression levels were normalized to *BcACT1* reference gene (Liu Y. et al., 2019). Data are means ± SD (*n* = 3). The double asterisks represent significant differences at *p* < 0.01 (Student’s t test).

FIGURE S2 **(A)** Diagram illustrating alignment of representative class I HDAC (HDAC1) and class II HDAC (HDAC6) in human with BcRPD3 and BcHda1 in the conserved regions containing AGG/VRPP, respectively. **(B)** qRT-PCR validation of *BcRPD3* gene overexpression in different strains. Expression levels were normalized to β*-tubulin* gene. Data are means ± SD (*n* = 3). **(C)** Immunoblot for the detection of BcRPD3-GFP in extracts of B05.10, three BcRPD3-GFP transformants and three BcRPD3-VRPP-GFP transformants using anti-GFP. Histone H3, which served as a loading control, was detected using anti-H3 antibody. **(D)** Statistical analysis of H3 and H4 acetylation. Data are means ± SD (*n* = 3).

FIGURE S3 **(A)** Distribution of functional classification of upregulated genes in BcRPD3-1. Histograms indicate *P-*values of the enriched functional categories. **(B)** RNA-seq analysis of 6 most significantly upregulated genes. Differential expression in three biological replicates is illustrated using a heat map with colored squares indicating the range of expression levels referred to log2 FPKM value. **(C)** qRT-PCR validation of downregulated genes. Expression levels were normalized to β*-tubulin* gene. Data are means ± SD (*n* = 3).

TABLE S1 Primers used in the study.

TABLE S2 Mapping statistics of the RNA-seq data.

TABLE S3 The RNA-seq analysis data of gene expression.
