# Supplementary figures and images for: BcRPD3-Mediated Histone Deacetylation Is Involved in Growth and Pathogenicity of Botrytis cinerea
Source: Front Microbiol. 2020 Jul 29;11:1832. doi: 10.3389/fmicb.2020.01832 (PMC7403187; doi:10.3389/fmicb.2020.01832)

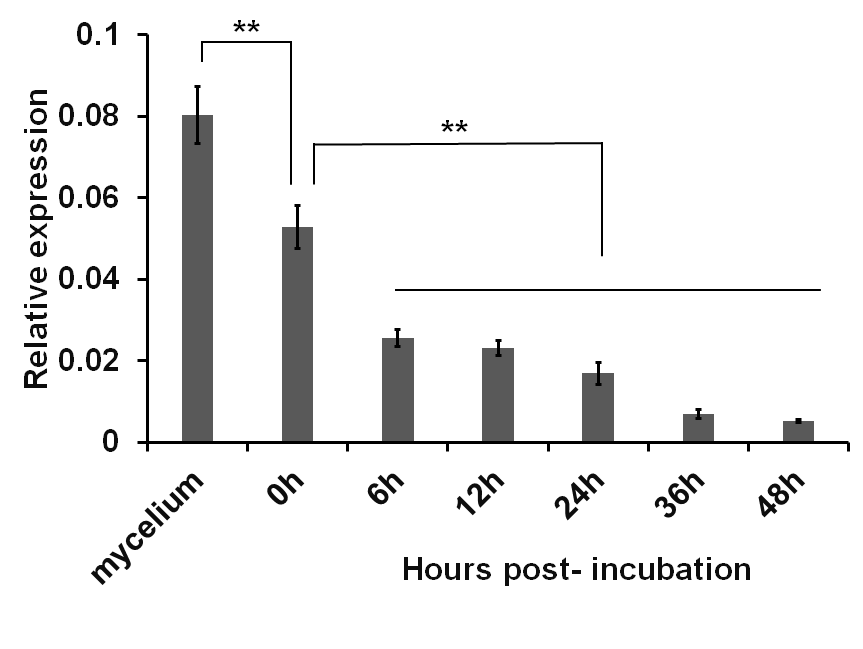

Supplement: FIGURE S1 — Expression profile of BcRPD3 in mycelium and during host infection by the pathogen. Expression levels were normalized to BcACT1 reference gene (Liu Y. et al., 2019). Data are means ± SD (n = 3). The double asterisks represent significant differences at p < 0.01 (Student’s t test). [file Image_1.TIF]

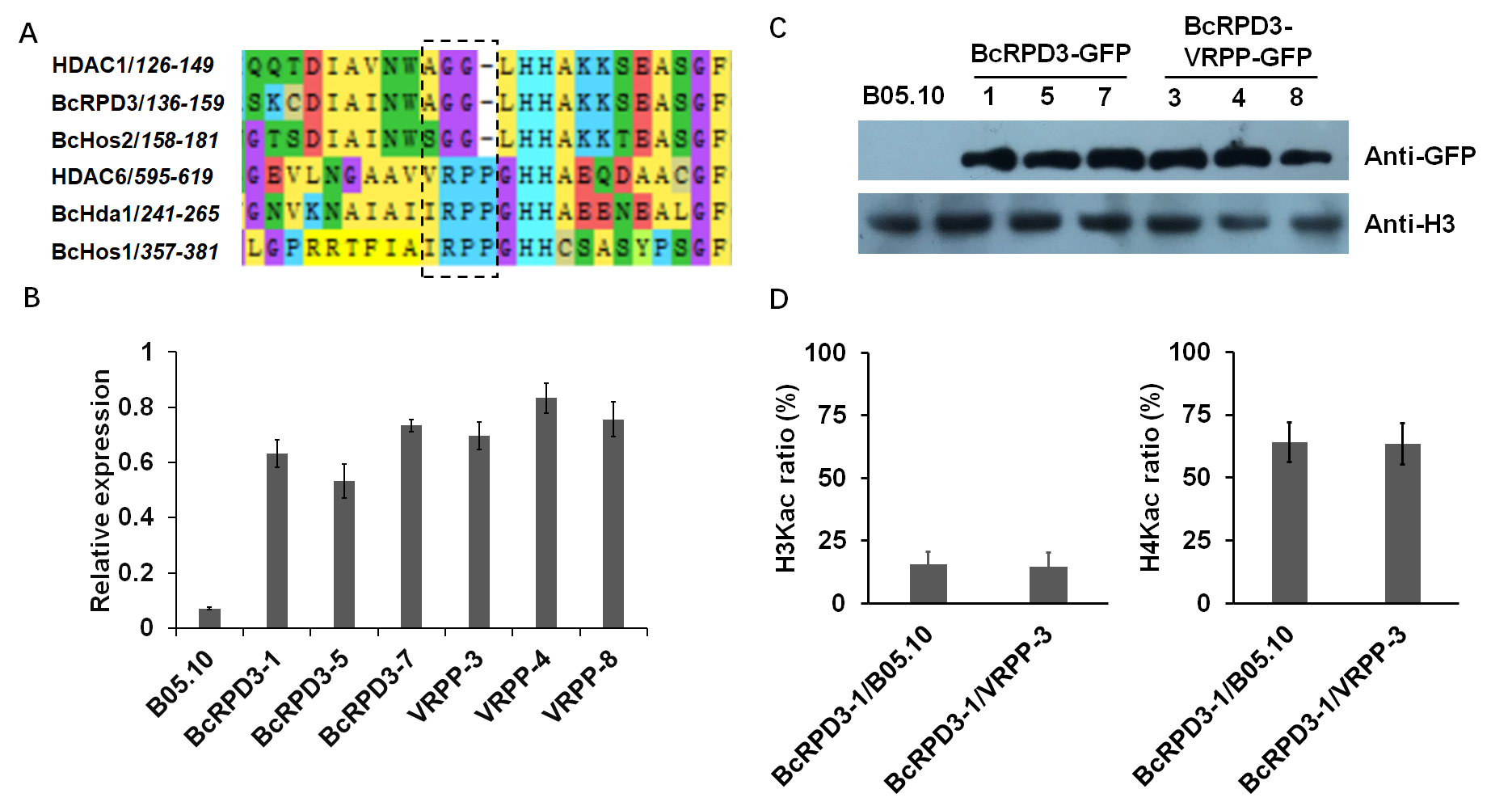

Supplement: FIGURE S2 — (A) Diagram illustrating alignment of representative class I HDAC (HDAC1) and class II HDAC (HDAC6) in human with BcRPD3 and BcHda1 in the conserved regions containing AGG/VRPP, respectively. (B) qRT-PCR validation of BcRPD3 gene overexpression in different strains. Expression levels were normalized to β-tubulin gene. Data are means ± SD (n = 3). (C) Immunoblot for the detection of BcRPD3-GFP in extracts of B05.10, three BcRPD3-GFP transformants and three BcRPD3-VRPP-GFP transformants using anti-GFP. Histone H3, which served as a loading control, was detected using anti-H3 antibody. (D) Statistical analysis of H3 and H4 acetylation. Data are means ± SD (n = 3). [file Image_2.TIF]

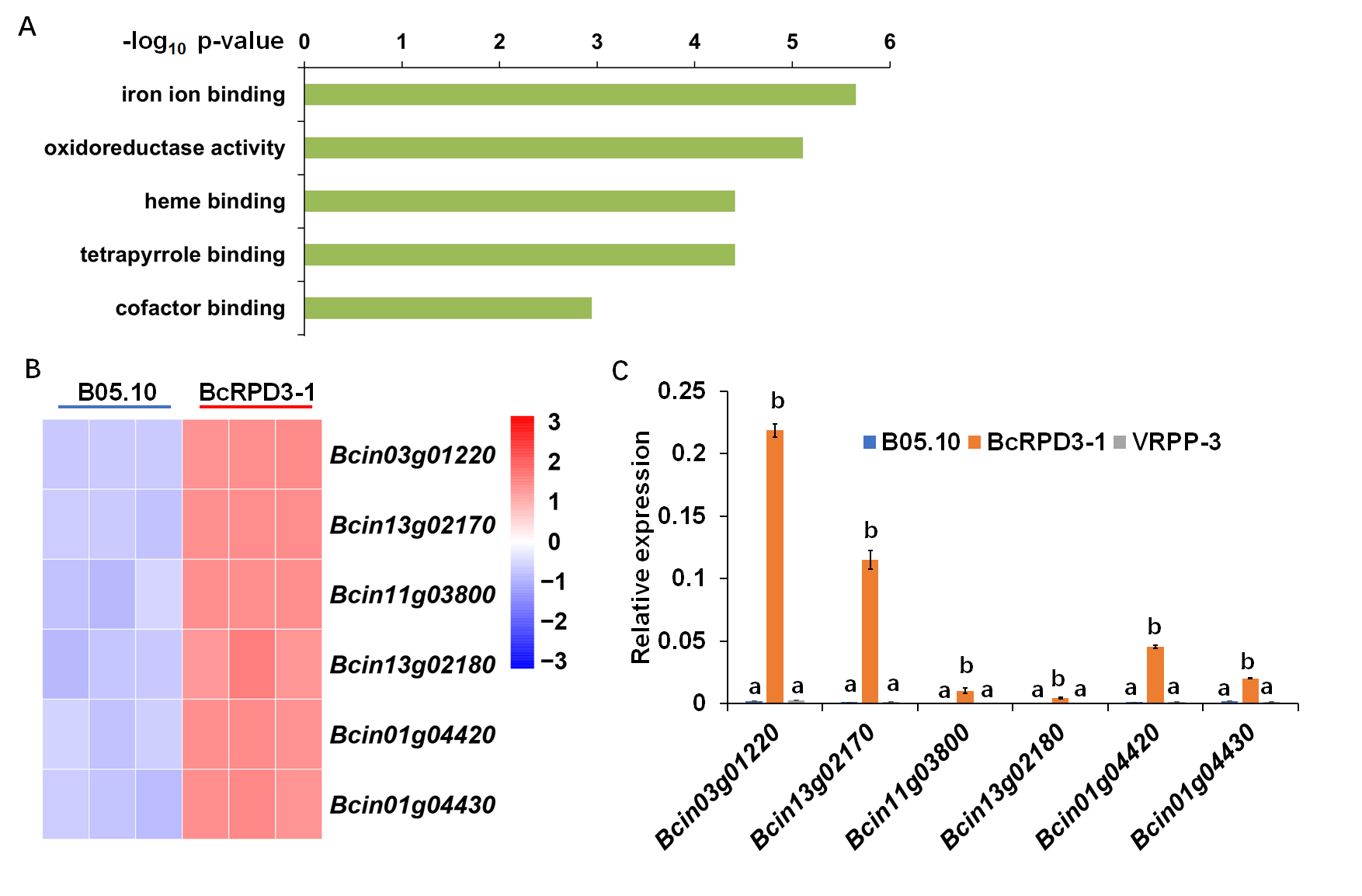

Supplement: FIGURE S3 — (A) Distribution of functional classification of upregulated genes in BcRPD3-1. Histograms indicate P-values of the enriched functional categories. (B) RNA-seq analysis of 6 most significantly upregulated genes. Differential expression in three biological replicates is illustrated using a heat map with colored squares indicating the range of expression levels referred to log2 FPKM value. (C) qRT-PCR validation of downregulated genes. Expression levels were normalized to β-tubulin gene. Data are means ± SD (n = 3). [file Image_3.TIF]
